# Pamphlets and Books Received

**Published:** 1884-02-20

**Authors:** 


### *PAMPHLETS & BOOKS RECEIVED.*

*The Proceedings of the Naval Medical Society*, with the following contents:

Double Aneurism of the Arch of the Aorta: Dr. Joseph H. Bryan, U. S. N.

Case of Oxalic Acid Poisoning: Dr. John C. Wise, U. S. N.

Hypertrophic Nasal Catarrh: Dr. Presley M. Rixey, U. S. N.

Yellow-fever on the U. S. S. Iroquois, 1883: Dr. J. W. Ross, U. S. N.

Conclusions as to the outbreak of Yellow-fever at Pensacola in 1882: Dr. William Martin, U. S. N.

Notes on the Yellow-fever at Pensacola in 1883: Dr. Daniel M. Guiteras, U. S. N.

Remarks on Yellow fever: Dr. Adolph A. Hoehling, U. S. N.

Washington: Printed by Judd & Detweiler. 1884.

*A Monograph on Ergot*, its Nature, Action and Uses, by Oscar Oldberg, P.H.G., M.D., and Otto A. Wall, P.H.G., M.D.

*Borderland Psychiatric Records—Prodromal Symptoms of Psychical Impairment*, by C. H. Hughes, M.D., St. Louis, Lecturer on Nervous Diseases in St. Louis Medical College.

*The Opium Psycho-Neurosis—Chronic Miconism or Papaverism*, by C. H. Hughes, M.D., St. Louis.

*Bichloride of Methylene used in a Junker's Inhaler*, by Jno. H. McIntyre, A.M., M.D. St. Louis: Reprinted from the St. Louis Medical and Surgical Journal, October, 1883.

*Hydriodic Acid.* Additional Notes in the Treatment of Acute Inflammatory Rheumatism, Asthma, Chronic Bronchitis, Adenitis, etc. Dr. Churchill's Method of using the Hypophosphites in Phthisis, with detailed History of Four Cases in the Cavernous Stage, successfully treated by him. Physiogenic and Pathogenic effects of the Hypophosphites, and eonditions claimed by him to be necessary to their successful use in Phthisis. Second Edition.

*Some Recent Progress in Diseases of the Nervous System.* by Talbot Jones, M.D., St. Paul, Minn.

*Introductory Address* Delivered before the Medical Class of Dartmouth College, August 1st, 1883, by Louis Elsberg, A.M., M.D., Professor of Laryngology.

*School Hygiene*, by Charles J. Lundy, A.M., M.D., Professor of Diseases of the Eye, Ear and Throat, in the Michigan College of Medicine, Detroit, Mich. Read before the American Health Association, 1883.

*Infusion of Jequirity*, or Licorice Bean, in Inverterate Pannus, with a report of several successful cases, by Edward S. Peck, M.D., Surgeon to the Eye and Ear Dapartment of the Northeastern Dispensary; Visiting Surgeon to the Ophthalmic Division of the Charity Hospital, New York. New York: Trow's Printing and Bookbinding Company, 201–213 East Twelfth Street, 1884.

